# Review of Corneal Biomechanical Properties Following LASIK and SMILE for Myopia and Myopic Astigmatism

**DOI:** 10.2174/1874364101812010164

**Published:** 2018-07-27

**Authors:** Iben Bach Damgaard, Mohamed Reffat, Jesper Hjortdal

**Affiliations:** 1 Department of Ophthalmology, Aarhus University Hospital, Aarhus, Denmark.; 2 Mansoura Ophthalmic Hospital, Mansoura, Egypt

**Keywords:** Myopia, SMILE, LASIK, Myopia astigmatism, Corvis, Corvist ST

## Abstract

Worldwide, femtosecond Laser Assisted *In-situ* Keratomileusis (LASIK) is a well known and commonly used refractive technique, although Small Incision Lenticule Extraction (SMILE) has become increasingly popular since it was introduced in 2011. In LASIK, a corneal flap is cut with a microkeratome or femtosecond laser, followed by thinning of the stromal bed with excimer laser ablation. In SMILE, a minor intrastromal lenticule is cut with a femtosecond laser and subsequently removed through a small incision, leaving the anterior and strongest part of the cornea almost intact. Both LASIK and SMILE require cutting of corneal lamellae that may reduce the biomechanical stability of the cornea, with the potential risk of corneal iatrogenic ectasia as a severe complication. However, SMILE preserves the anterior corneal integrity and may, in theory, better preserve the corneal biomechanical strength than LASIK after surgery.

A review aimed to examine the current literature that describes and compares the corneal biomechanical properties after Laser Assisted *In-situ* Keratomileusis (LASIK) and Small Incision Lenticule Extraction (SMILE). A comprehensive search was performed in Pubmed.gov using the following search queries: Corneal biomechanical properties, corneal biomechanics, ocular response analyser, ocular response analyzer, ORA, *ex vivo, in vitro*, Corvis, Corvis ST, LASIK, and SMILE.

## 1 INTRODUCTION

During the last few decades, laser refractive surgery has gained extensive interest for correction of refractive errors such as myopia, hyperopia, astigmatism, and presbyopia. Worldwide, femtosecond laser-assisted in-situ keratomileusis (LASIK) has been widely implemented in clinical practice [1] In LASIK, a reproducible flap of a predetermined thickness is made with a femtosecond laser, and a stromal bed shaped with excimer laser according to the amount of refractive correction is needed. In 2011, Small Incision Lenticule Extraction (SMILE) was introduced as a refinement of the LASIK technique [2, 3]. In SMILE, an intrastromal lenticule is cut with a femtosecond laser, and subsequently removed through a minor incision. Both SMILE and LASIK have shown high efficacy, predictability, and safety [4-9], but SMILE may have an advantage of being a flap-free procedure preserving the corneal biomechanical strength better than LASIK [10, 11].

The cornea consists of approximately 200 collagen lamellae containing collagen fibres that are crucial to withstand the intraocular pressure and maintain the corneal shape [12, 13]. The lamellar interweaving and corneal cross-linking ensure the biomechanical strength of the cornea, the anterior 1/3 being the strongest part [14]. The tensile strength in the corneal lamellae decreases after incision and does not contribute to the overall corneal resistance to the intraocular pressure. Due to the orientation of the lamellae, a vertical cut causes greater reduction in the corneal biomechanical strength than a cut parallel to the corneal surface. [15]. The irreversible corneal alterations after refractive surgery affect the biomechanical properties by reducing the biomechanical strength. Thus, iatrogenic ectasia is one of the severe complications caused by a biomechanical weakening after laser refractive surgery, seen by corneal thinning, protrusion, increased myopia, irregular astigmatism, and decreased visual acuity [16]. Iatrogenic ectasia has been reported in a few cases after both LASIK [17] and SMILE [18-21], although the comprehensive evaluation of the SMILE cases showed preoperative abnormal topographic patterns in almost all cases.

LASIK may cause a greater reduction of the biomehcanical strength due to the almost circumferential cut during flap creation, compared with only a 2-3 mm incision length in SMILE. Several studies have tried to answer this question both **in vivo** and *ex vivo,* and by mathematical or finite-element models of the cornea. This review aimed to describe current literature regarding corneal biomechanical properties after LASIK and SMILE. Our search was performed in pubmed.gov, using the following research terms: Corneal biomechanical properties, corneal biomechanics, ocular response analyser, ocular response analyzer, ORA, *ex vivo*, *in vitro*, Corvis, Corvis ST, LASIK, and SMILE. A total of 57 studies were included in the initial search, where six studies were excluded due to non-English language. Based on abstract reading, 14 studies described and compared the biomechanical properties following LASIK and SMILE. Comparisons with other laser refractive techniques such as photorefractive keratectomy (PRK), FLEX, and LASEK as well as the dependency of IOP, CCT, refractive status, cap/flap thickness, and age were outside the topic of this review.

### 1.1 *In vivo* Corneal Biomechanical Assessment

Several approaches to *in* vivo assessment of the biomechanical properties have been proposed, including Brillouin microscopy [22], optical coherence elastrography [23], and supersonic shear wave elastrography [24]. To date, there are only two commercially available devices to analyse some corneal biomechanical properties **in vivo**; the Ocular Response Analyser (ORA) [25] and the Corvis ST [26].

#### 1.1.1 The Ocular Response Analyser

The Ocular Response Analyser (ORA, Reichert Inc., Dephew, NY) is a non-contact differential tonometer evaluating the *in vivo* corneal viscoelasticity and intraocular pressure during a collimated air-pulse pressurizing the corneal apex [25]. The corneal inward (P1) and outward (P2) applanation points are registered with an electro-optical infrared system Fig. (**1**) and translated into IOP values based on the applied pressure. Corneal Hysteresis (CH) is defined as the difference between the applied pressure during inward and outward applanation (CH=P1-P2), and describes cornea ability to dissipate energy due to viscous damping. Corneal hysteresis reflects the combined change in the viscosity and elasticity, previously shown in an experimental study with the ORA [27]. Corneal Resistance Factor (CRF) is determined by an empirical formula, based on the correlation between P1, P2, and CCT, reflecting the overall corneal resistance. Both values have been shown to be affected by CCT [28-31], IOP [30-32], and age [33-35], and must be taken into consideration when interpreting ORA outcomes.

#### 1.1.2 The Corvis ST

(Oculus, Wetzlar, Germany) was later presented as an alternative device for *in vivo* acquisition of the corneal biomechanical properties. It combines non-contact tonometry with high-speed Scheimpflug visualization of the corneal deformation during the symmetrically metered air pulse Fig. (**2**). With 4330 frames per second, the Corvis ST records the dynamic deformation and determines velocity, length, and time lapse during applanation and highest concavity Table **1**. The initial Corvis ST software version presented a limited number of parameters; later new variables followed describing the inward and outward applanation in further detail.

### 2 THE ORA: LASIK VERSUS SMILE

Several studies have assessed the ORA parameters after laser refractive procedures to describe corneal biomechanical properties after both LASIK and SMILE, whereas only a few studies have compared the biomechanical alterations after surgery Table **2** [26, 36-44]. A retrospective study by Osman *et al.* [38] examined 25 LASIK- and 25 SMILE-treated patients one month after surgery, and found a more profound reduction in the CH and CRF parameters after LASIK than SMILE (preop SE: LASIK −5.16±1.42D, SMILE −5.43±1.17D). These findings were in agreement with Wang *et al.* [42] where CH and CRF decreased more in LASIK-treated patients with myopia more than -6D at one week, and one and three-month follow-up. However, the authors did not find any difference in the CH or CRF reduction in patients treated for less than -6D. Wang *et al.* [42] also examined the additional p1 and p2 areas, describing the area of the amplitude during inward and outward applanation. In their comparative study of 79 LASIK- and 187 SMILE- treated patients, the p1 and p2 areas decreased more after LASIK than after SMILE in patients needing more than -6D correction, suggesting a generally softer cornea after LASIK than after SMILE [45]. However, the difference was non-significant in their study group of low myopic patients.

The individual variation in corneal biomechanical properties may cause an in-between group difference when comparing the ORA parameters. Hence, a paired-eyed study design provides more strength when evaluating the biomechanical properties after laser refractive surgery. Agca *et al.* [44] examined in a prospective paired-eyed study patients treated with LASIK or SMILE in each of the two eyes (preop SE: LASIK −3.71±1.83D, SMILE −3.62±1.79D), and found a similar reduction in viscoelasticity at six months. However, this may be caused by correction of moderate to low myopia with only a minor reduction in the corneal stiffness beyond the sensitivity of the device [46]. High myopic correction requires removal or ablation of more stromal tissue than low myopic correction. Thus, Li *et al.* [37] examined the average decrease of CH and CRF per amount of removed or ablated tissue, and found a greater reduction after LASIK than SMILE (preop SE: LASIK -5.95±1.78D, SMILE -5.60±1.43D), which may be attributed to the flap creation during LASIK.

A major limitation of a few of the comparative studies was the lack of corneal-compensated IOP and CCT comparisons between groups, as these parameters in some studies were correlated with both CH and CRF [28, 30]. Pedersen *et al.* [26] evaluated estimated marginal means in 35 LASIK- and 29 SMILE-treated patients and took into consideration the dependency of IOP and postoperative CCT. No significant differences were found in CH and CRF up to one year after surgery, although the preoperative values were not reported in this study. However, both CH and CRF describe the viscoelasticity of the corneal tissue, where an increase in elasticity may equalize the decrease in viscosity and thereby mask an actual impact of the surgical intervention on the elasticity [27].

## 3 THE CORVIS ST: LASIK VERSUS SMILE

Shen *et al.* [47] were the first to retrospectively report the biomechanical outcomes after LASIK and SMILE using the Corvis ST. They found no significant differences in any of the evaluated parameters three months after surgery Table **3**. However, only the postoperative values were described, whereas a comparison of the average change due to surgery would provide more information about the biomechanical impact following LASIK and SMILE. Sefat *et al.* [48] also reported similar biomechanical responses after LASIK and SMILE with the Corvis ST in a subgroup matched for age, preoperative CCT, IOP, preoperative spherical equivalent, and ΔCCT. Osman *et al.* [38] calculated and compared the percentage of change in preoperative and postoperative measurements in a comparative study of 25 LASIK- and 25 SMILE-treated patients. The authors found significant less reduction in A1 time, HC time, and A2 time after SMILE than LASIK, which may reflect a less compliant cornea after the flap-free procedure. Furthermore, the percentage of increase in deformation amplitude during highest concavity was significantly larger in LASIK than SMILE, suggesting a more severe inward deformation during the air pulse after LASIK, possibly due to a more compliant cornea.

It has previously been questioned if the repeatability and reproducibility of the Corvis ST parameters available with the first software version were acceptable (Table **2**) [49-51]. Hence, a retrospective study by Pedersen *et al.* [26] examined only the variables with a coefficient of variation <10% [49] including A1 deflection length and HC deflection length, which were not standard parameters in the initial Corvis ST software. After adjusting for postoperative CCT, IOP, and age, only HC Time was significantly shorter in LASIK than SMILE, suggesting that a LASIK- treated corneas reached their highest concavity at an earlier stage. However, the shorter HC time was not seen in an additional group of FLEX-treated patients, a refractive technique also requiring the creation of a corneal flap (Femtosecond Lenticule Extraction). Furthermore, none of the remaining Corvis ST parameters supported the hypothesis of a more compliant cornea after LASIK compared with SMILE [26].

### 3.1 *Ex vivo* Corneal Biomechanical Assessment

Only limited peer-reviewed *ex vivo* studies have assessed and compared the biomechanical weakening after cap and flap creation. A study by Cartwright *et al.* [15] examined the corneal compliance after femtosecond lamellar and side cut in human donor corneas mounted on artificial anterior chambers. With radial shearing speckle pattern interferometry, the authors calculated the corneal apical displacement during increased chamber pressure (inflation test). They found a higher corneal compliance after side cut incision than after in-plane delamination, due to more severe damaging of the collagen fibres. Thus, the average percentage increase in apical displacement during inflation was 5% for delamination in 160μm depth and 33% after 90° side cut in 160μm depth. Although these results suggest more severe weakening after flap creation than after pocket creation, the authors did not examine the effect of a minor incision created using SMILE.

Traditional strip extensiometry was also used to examine the biomechanical properties after LASIK and SMILE presented by Kanellopoulos *et al.* (ePoster PA049, AAO, 13^th^ November 2015, Las Vegas). The biomechanical tensile strength was examined in four laser refractive groups treated with LASIK and SMILE for -3D and -8D, respectively. Using biaxial in-plane tensile tests on laser-treated corneas, the authors found a similar reduction in tensile strength in LASIK and SMILE for higher myopic corrections (-8D), but less tensile strength reduction in LASIK than SMILE for the low myopic group (-3D). One possible explanation may be the surgical approach, as SMILE requires removal of more tissue than ablated in LASIK to reach equivalent correction. However, these results should be interpreted with caution as the orientation of the collagen fibrils and the pressure induction in strip extensiometry are not similar to the intact eye. Furthermore, another and similar *ex* vivo strip extensiometry study of porcine eyes treated with SMILE and FLEX suggests that SMILE may be superior in terms of biomechanical stability after surgery [52].

## 4 FINITE-ELEMENT 3D MODELS OF STRESS DISTRIBUTION

Corneal biomechanical alterations after LASIK and SMILE have previously been compared in a computer modelling study using a finite-element anisotopic collagen fibre-dependent model [10]. By tomographic measurements from a normal, a LASIK-treated, and a SMILE-treated cornea, the authors managed to compare the stress distribution after SMILE and LASIK with a geometric analogue model using an untreated control cornea. The stress distribution in the SMILE simulation was comparable to the analogue model with a maximal stress in the superficial layer of the cornea. For the LASIK simulation, the stress was greater in the residual stromal bed after LASIK compared with the corresponding geometry analogue. A simulated thicker flap in LASIK caused greater increase in the corneal stress values than removal of a SMILE lenticule in the deeper layer. Thus, the study suggests that the stromal residual bed after LASIK is exposed to increased stress due to the flap creation, while SMILE preserves the biomechanical strength almost similar to what is seen in an untreated cornea [10].

## 5 MATHEMATICAL MODEL OF TENSILE STRENGTH DISTRIBUTION

Cohesive strength [14], tangential tensile strength [53], and shear strength [54] have been shown to decrease through the cornea with the anterior 40% being the strongest part. Consequently, Reinstein *et al.* [11] developed a mathematical model of the non-linear tensile strength distribution in the cornea to evaluate the biomechanical weakening after LASIK and SMILE. Data was retrieved from a previous **in vitro** strip extensiometry study on evaluation of the corneal stromal tensile strength as a function of depth [14]. The model predicted that the postoperative total stromal strength was better preserved after SMILE than after LASIK due to the creation of a corneal cap rather than a flap. Hence, if 100-μm tissue removal was performed in a 550-μm thick cornea, the postoperative relative total tensile strength would be 75% after SMILE (130-μm cap) and 54% after LASIK (110-μm flap). Furthermore, the model predicted that an extracted SMILE lenticule approximately 100 μm thicker than the ablation depth in LASIK would provide the same postoperative reduction in the total tensile strength (130-μm cap and 110-μm flap). Depending on the ablation and lenticule profile, this would correspond to a possibility for 7.75D more myopic correction in SMILE than LASIK with equivalent weakening of the postoperative total strength. It may be questioned, if it is actually possible to correct higher degrees of myopia with SMILE than LASIK, using a residual bed thickness below the generally recommended 250 μm limit [11].

## CONCLUSION

Corneal biomechanical properties are of major importance in laser refractive surgery and must be taken into consideration to reduce the risk for iatrogenic ectasia. Some **in vivo** corneal biomechanical alterations are possible to quantify with ORA and Corvist ST but are difficult to interpret and use in clinical practice due to their dependence of IOP, CCT, refractive status, and age. Development and refinement are needed if ORA and Corvis ST should be implemented in a screening procedure of the biomechanical strength before refractive surgery. Mathematical analysis and finite-element models suggest that SMILE may preserve corneal biomechanical properties better than LASIK. Furthermore, the current **in vivo** studies performed with ORA and Corvis ST indicate that SMILE is equal or superior to LASIK in terms of preservation of the postoperative biomechanical strength. However, future paired-eyed studies comparing LASIK and SMILE are needed to support this conclusion and to eliminate the in-between group variability in corneal biomechanical properties. As iatrogenic ectasia has been reported in very few patients, it is recommended to follow the same contraindications as used in LASIK, especially when planning SMILE in borderline or corneas at risk.

## Figures and Tables

**Fig. (1):**
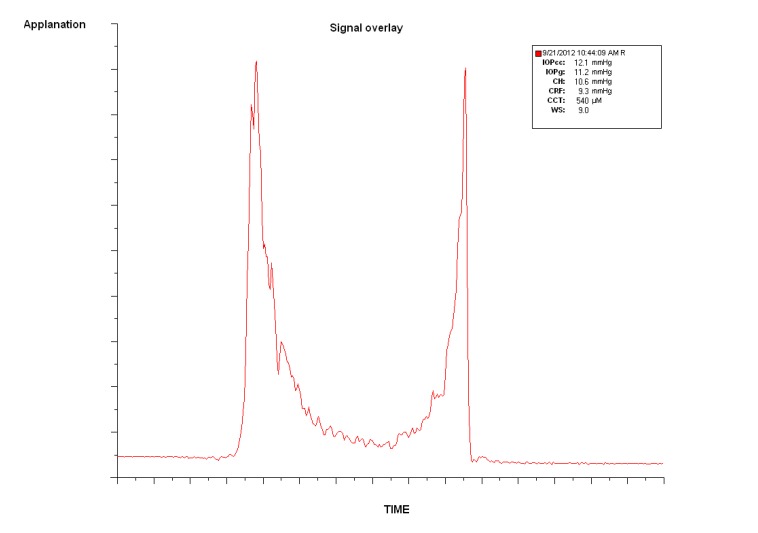
Screenshot from the Ocular Response Analyser showing the applanation signal at inward and outward applanation (red peaks). Corneal Hysteresis (CH) is defined as the difference in the applied pressure during the first and second applanation. CRF: Corneal Resistance Factor. IOPg: Goldman Correlated Intraocular Pressure. IOPcc: Corneal Compensated Intraocular Pressure. CCT: Central Corneal Thickness.

**Fig. (2):**
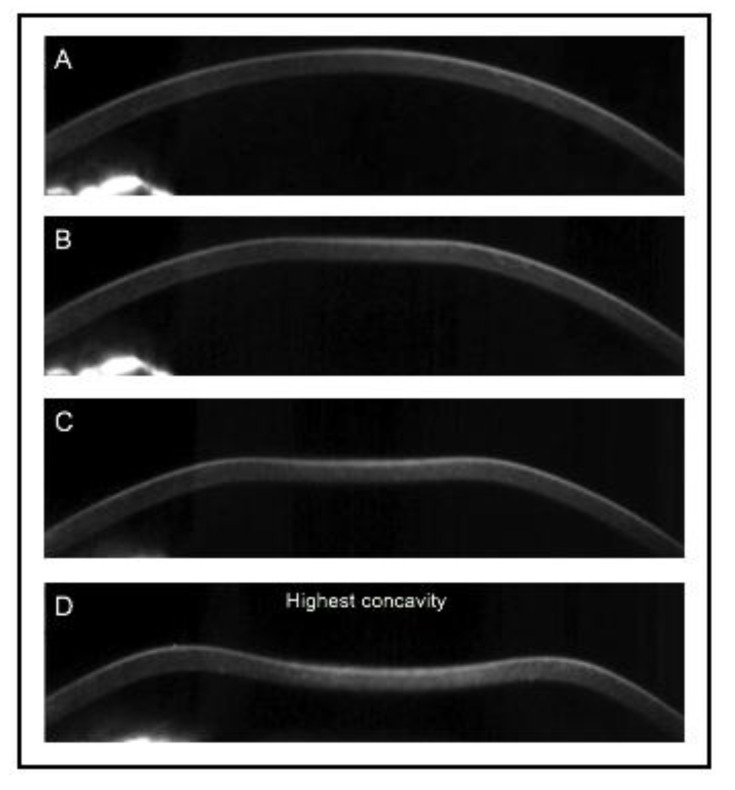
Screenshot of corneal deformation during an air-pulse from the Corvis ST with first inward applanation and maximal deformation.

**Table 1:** Description of Corvis ST parameters. *: Parameters avaliable with the first software version.

**Corvis ST parameters**	**Description**
IOP [mmHg]*	The Intraocular pressure, calculated from A1
Pachymetry [μm] *	Central corneal thickness, measured with optical pachymetry
A1 time [ms] *	Time to first (inward) applanation
A1 length [mm]*	Length of first (inward) applanation
A1 velocity [m/s]*	Velocity of the corneal apex at first applanation
**A1 deformation amplitude [mm]**	Sagittal deformation length of the apex at first applanation
**A1 deflection length [mm]**	Horizontal length of the deformed part of the cornea at first applanation
**A1 deflection amplitude [mm]**	Deformation amplitude corrected for whole eye movement at first applanation
HC deformation amplitude [mm] *	Sagittal deformation length of the apex at highest concavity
HC time [ms] *	Time to reach highest concavity
HC radius [mm] *	Radius of curvature at highest concavity, calculated with “parabolic fit”
**HC deflection length [mm]**	Horizontal length of the deformed part of the cornea at highest concavity
**HC deflection amplitude [mm]**	Deformation amplitude corrected for whole eye movement at highest concavity
**HC deflection amplitude [ms]**	Time of highest concavity deflection amplitude
Peak distance [mm] *	Distance between peak points at highest concavity
A2 time [ms] *	Time to reach second (outward) applanation
A2 length [mm] *	Length of second (outward) applanation
A2 velocity [m/s] *	Velocity of the corneal apex at second applanation
**A2 deformation amplitude [mm]**	Sagittal deformation length of the apex at second applanation
**A2 deflection length [mm]**	Horizontal length of deformed part of the cornea at second applanation
**A2 deflection amplitude [mm]**	Deformation amplitude corrected for whole eye movement at second applanation

**Table 2:** Studies comparing ORA measurements following LASIK and SMILE. Re com: Retrospective comparable study. Pro com: prospective comparable study. RCT paired: Randomized, controlled, paired-eyed study. * graphical illustration, but values not reported. ** Standard derivations not reported in text. n: number of eyes. CH: Corneal Hysteresis. CRF: Corneal Resistance Factor. Δ: postoperative – preoperative.

-			**Preoperative**							**Postoperative**				-
			**LASIK**				**SMILE**			**LASIK**		**SMLE**		
**Study**	**Design**	**n**		**CH [mmHg]**	**CRF [mmHg]**		**n**	**CH [mmHg]**	**CRF [mmHg]**	**CH [mmHg]**	**CRF [mmHg]**	**CH [mmHg]**	**CRF [mmHg]**	**Conclusion**
**Xia *et al.* 2016 **[36]	Pro com	59		10.76 ± 1.67	10.60 ± 1.99		69	10.99 ± 1.65	11.26 ± 1.94	1W: 7.80 ±1.57 1M: 7.76 ±1.21 3M: 8.06 ±1.06 6M: 7.97 ±1.14	1W: 7.14 ±1.94 1M: 6.51 ±1.33 3M: 6.53 ±1.38 6M: 6.31 ±1.41	1W: 7.82 ±1.32 1M: 8.47 ±1.23 3M: 8.35 ±1.08 6M: 8.58 ±1.40	1W: 7.57 ±1.44 1M: 7.09 ±1.53 3M: 6.51 ±1.27 6M: 7.05 ±1.65	A significant reduction was seen in CH and CRF in both groups. At 6 months, no difference were seen in CH between groups (p=0.052), while the difference in CRF was significant (p=0.023)
**Li *et al.* 2016**[37]	Re com	96		10.32	10.74		97	10.16	10.41	1M: 7.48 3M: 7.74 6M: 7.84	1M: 6.93 3M: 6.70 6M: 6.58	1M: 7.82 3M: 7.99 6M: 7.94	1M: 7.06 3M: 6.82 6M: 6.83	ΔCRF and ΔCH per removed or ablated tissue were higher in LASIK than in SMILE. **
**Osman *et al.* 2016**[38]	Re com	25		11.59±1.86	11.00 ± 1.89		25	12.03 ± 1.76	11.42 ± 1.68	1M: 8.46±1.76	1M: 7.45±2.39	1M: 9.99±1.76	1M: 9.43±1.55	The average reduction in CH and CRF (in percentage) was significantly larger after LASIK than SMILE at one month (p<0.001)
**Zhang *et al.* 2016**[39]	Pro com	80		10.83±1.60	10.71 ± 1.74		80	10.64 ± 1.09	10.54 ± 1.53	24H: 7.98±1.17 2W: 8.07±1.37 1M: 8.17±1.31 3M: 8.00±1.32	24H: 6.85±1.42 2W: 6.87±1.45 1M: 6.88±1.46 3M: 6.82±1.40	24H: 7.91±1.06 2W: 7.94±1.08 1M: 8.00±0.99 3M: 7.91±0.92	24H: 6.88±1.47 2W: 7.01±1.38 1M: 7.08±1.34 3M: 7.07±1.27	ΔCH and ΔCRF did not differ between WF-guided LASIK and SMILE at any postoperative time points
**Pedersen *et al.* 2015**[26]	Re com	35		n/a	n/a		29	n/a	n/a	37M: 8.58±0.15	37M: 7.12±0.18	15M: 8.56±0.19	15M: 7.12±0.23	Reported estimated marginal means (36.7 years, 473μm, IOPcc 13.0mmHg). No significant differences in CH and CRF between LASIK and SMILE
**Wang *et al.* (2016) **[40]	Re com	56		10.85 ±1.19	10.62 ±1.81		50	10.52 ±1.71	10.07 ±1.49	6M: 8.43±1.75 12M: 8.31±1.62	6M: 7.53 ±1.81 12M: 7.29 ±1.76	6M: 7.85±1.81 12M: 7.97±2.05	6M: 7.54±1.66 12M: 7.83±1.64	Corneal biomechanical changes were similar after the two procedures, although FS-LASIK demonstrated a greater reduction in CRF.
**Wu & Wang (2015) **[41]	Re com	75		10.09±1.38		10.57±1.64	75	10.16±1.30	10.39±1.52	3M: 7.86±1.03	M3: 6.77±1.13	M3: 8.30±1.04	M3: 7.25±1.31	Postoperative CH and CRF were significantly higher after SMILE than after FS-LASIK (p<0.015)
**Wang *et al.* 2014**[42]	pro com	79		High myopia: 10.15±0.27 Low myopia: 10.45±0.19	High myopia: 10.15±0.31 Low myopia: 10.07±0.20		187	High myopia: 10.49 ± 0.19 Low myopia: 10.56 ± 0.17	High myopia: 10.86 ± 0.20 Low myopia: 10.48 ± 0.17	1W: n/a * 1M: n/a * 3M: n/a *	1W: n/a * 1M: n/a * 3M: n/a *	1W: n/a * 1M: n/a * 3M: n/a *	1W: n/a * 1M: n/a * 3M: n/a *	High myopia: CH and CRF decreased significantly more after LASIK than after SMILE (p<0.014). Low myopia: No significant difference between LASIK and SMILE
**Wu *et al.* 2014**[43]	Pro com	40		n/a	n/a		40	n/a	n/a	1W: n/a 3M: 8.17±0.71 6M: 8.11±0.66	1W: 7.21±0.83 1M: 7.29±0.75 6M: 6.94±0.66	1w: n/a 3M: 8.64±1.03 6M: 8.59±1.00	1W: 7.89±1.31 1M: 7.98±1.24 6M: 7.78±1.03	Average ΔCRF was significantly larger after LASIK than SMILE at six months (p=0.025). Average ΔCH was comparable at any time points (p=0.083)
**Agca *et al.*** **2014 **[44]	RCT paired	30		11.00±1.53	10.76±1.45		30	10.89±1.79	10.73±1.71	1M: 8.80±1.51 6M: 9.02±1.27	1M: 7.98±1.58 6M: 8.07±1.26	1M: 8.70±1.31 6M: 8.95±1.47	1M: 7.89±1.57 6M: 7.77±1.37	Average ΔCH and ΔCRF was similar between LASIK and SMILE at one and six months follow up. The difference in CH and CRF between one and six months was comparable in LASIK and SMILE

**Table 3:** Studies comparing Corvis ST parameters following LASIK and SMILE. Only the original Corvis ST values are included in the table. * Estimated marginal means at following values: Age at examination 36.7 years, CCT 472 mm, IOPcc 13.0mmHg. HC PD: Highest concavity peak distance. HC DA: Highest concavity deformation amplitude.

		**Sefat *et al.* 2016** [48]		**Pedersen *et al.* 2015** [26]		**Osman *et al.* 2015** [38]		**Shen *et al.* 2014** [47]	
	**Design**	Prospective, comparable		Retrospective, comparable		Retrospective, comparable		Retrospective, comparable	
		**Preop**	**Postop**	**Preop**	**Postop**	**Preop**	**Postop**	**Preop**	**Postop**
**LASIK**	**No of eyes**	48	48	n/a	35	25	25	17	17
	**Follow up**		3 months		37months		1 month		3 months
	**A1 Time**	7.34±0.41	6.84±0.21	n/a	6.82±0.02*	8.40±0.39	7.89±0.44	n/a	7.17±0.17
	**A2 Time**	21.65±0.44	22.04±0.31	n/a	21.7±0.06*	23.42±1.20	20.28±1.87	n/a	22.92±0.82
	**A1 length**	1.79±0.25	1.79±0.36	n/a	n/a	2.10±0.23	1.93±0.23	n/a	1.73±0.30
	**A2 length**	1.86±0.47	1.61±0.59	n/a	n/a	1.90±0.24	1.81±0.21	n/a	1.33±0.48
	**A1 velocity**	0.15±0.03	0.15±0.04	n/a	n/a	n/a	n/a	n/a	0.12±0.03
	**A2 velocity**	-0.38±0.11	-0.53±0.12	n/a	n/a	n/a	n/a	n/a	-0.53±0.12
	**HC Time**	16.71±0.48	16.79±0.47	n/a	16.1±0.08*	17.74±0.71	14.40±1.27	n/a	17.57±0.83
	**HC Radius**	8.18±1.31	7.07±0.77	n/a	6.06±0.09*	7.69±1.14	7.00±1.06	n/a	6.30±1.41
	**HC PD**	4.83±0.82	5.32±0.53	n/a	n/a	3.81±0.49	4.90±0.67	n/a	5.74±0.28
	**HC DA**	1.04±0.12	1.11±0.10	n/a	1.15±0.02*	1.02±0.10	1.26±0.07	n/a	1.19±0.13
**SMILE**	**No of eyes**	80	80	n/a	29	25	25	17	17
	**Follow up**		3 months		15 months		1 month		3 months
	**A1 Time**	7.25±0.33	6.74±0.25	n/a	6.75±0.03*	8.40±0.36	8.23±0.37	n/a	7.27±0.20
	**A2 Time**	21.73±0.37	22.01±0.86	n/a	21.8±0.07*	23.64±1.03	22.03±1.11	n/a	23.08±0.44
	**A1 length**	1.79±0.24	1.71±0.34	n/a	n/a	2.10±0.22	1.90±0.20	n/a	1.74±0.32
	**A2 length**	1.84±0.48	1.46±0.53	n/a	n/a	1.90±0.20	1.75±0.20	n/a	1.67±0.64
	**A1 velocity**	0.15±0.03	0.14±0.03	n/a	n/a	n/a	n/a	n/a	0.13±0.03
	**A2 velocity**	-0.39±0.08	-0.56±0.20	n/a	n/a	n/a	n/a	n/a	-0.49±0.15
	**HC Time**	16.91±0.42	16.81±0.35	n/a	16.4±0.01*	18.39±0.92	16.32±1.10	n/a	17.38±0.81
	**HC Radius**	7.89±0.82	6.60±0.68	n/a	6.25±0.11*	7.99±1.35	6.91±1.25	n/a	5.74±0.91
	**HC PD**	4.46±1.10	5.37±0.59	n/a	n/a	4.09±0.69	4.72±0.71	n/a	5.57±0.41
	**HC DA**	1.05±0.09	1.13±0.10	n/a	1.20±0.01*	1.05±0.08	1.10±0.08	n/a	1.17±0.11
	**Conclusion**	In a subgroup matched for spherical equivalent (26 LASIK, 43 SMILE), no significant differences were found in the postoperative Corvis ST parameters		HC time was significantly shorter after LASIK compared with SMILE, while no differences were seen in remaining parameters		The percentage of reduction in A1 Time, HC Time and A2 time was larger after LASIK than SMILE. The percentage of increase in HC peak distance and deformation amplitude was significantly larger after LASIK than SMILE.		No significant differences in mean values of deformation amplitudes and time between LASIK and SMILE.	
